# Reproduction and production in a social context: Group size, reproductive skew and increasing returns

**DOI:** 10.1111/ele.14157

**Published:** 2023-01-05

**Authors:** Ronald Lee, C. Y. Cyrus Chu

**Affiliations:** ^1^ Graduate School in Demography and Economics Berkeley California USA

## Abstract

Evolutionary success requires both production (acquisition of food, protection and warmth) and reproduction. We suggest that both may increase disproportionately as group size grows, reflecting ‘increasing returns’ or ‘group augmentation benefits’, raising fitness in groups that cooperate in production and limit reproduction to one or a few high fertility females supported by non‐reproductives, with high reproductive skew. In our optimisation theory both Allee effects (when individual fitness increases with group size or density) and reproductive skew arise when increasing returns determine optimal group size and proportion of reproductive females. Depending on which of food or maternal time is more important for reproduction, evolutionary trajectories of lineages may (1) reach a boundary constraint where only one female reproduces in a period (as with African wild dogs) or (2) reach a boundary where all females reproduce during their lifetimes but only during an early life stage (human menopause) or a late life stage (birds with non‐dispersing helpers), where stage length optimises the proportion of females that is reproductive at any time or (3) reach the intersection of these boundary constraints where a single reproductive female is fully specialised in reproduction (as with eusocial insects). We end with some testable hypotheses.

## Graphical abstract

A female's average cost per birth declines as her number of births rises, so a cooperative group achieves more births by limiting reproduction to a single female with others supporting. This reproductive skew benefits the group and generates high relatedness within it, raising indirect fitness benefits. Direct benefits arise through increasing returns to group ‘production’ of food, protection and warmth. Evolution can lead to a single lifetime reproductive female or to stage‐based cooperative reproduction. p: proportion of females in group that are reproductive. x: degree of specialization of reproductive females in reproduction. Line indicates evolutionary trajectory from solitary to stage‐based reproductive limitation in group.

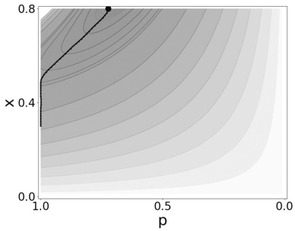

## INTRODUCTION

Sociality has been broadly defined as ‘cooperative group living’ which includes only about 2% of insect species, 5% of mammals and 9% of birds (Rubenstein & Abbot, 2017a), yet social animals account for at least half the biomass of all animals (Hölldobler & Wilson, 2009). Here, we develop a formal theoretical model to characterise the benefits of some strong forms of sociality that include cooperative breeding with reproductive suppression and high reproductive skew. Our model is abstract and does not correspond exactly to any particular species or lineage.

A central feature of our approach is that cooperative social groups ‘produce’ benefits which we call ‘outputs’ subject first to increasing then diminishing returns to group size. We do not intend anything new here except perhaps the terms used and reference to group aggregates. Standard lists of benefits or outputs in the literature include acquisition of food, predator defence, thermal regulation, information sharing and defence of foraging range. Similarly, costs of cooperative group living include increased competition, difficulties in recognising group members and increased exposure to parasites and infectious diseases. Ideally, a comprehensive measure of total net output would be formulated as an appropriately weighted sum of these components but that is not feasible (Creel & Creel, 2002:95).

Production of total output or any of its components may exhibit ‘increasing returns’ in the sense that enlarging group size by x% would raise net output by more than x%. However, beyond some size increasing returns give way to diminishing returns. ‘Models of group foraging assume that the marginal benefits of group size decrease with increasing group size, whereas the costs of grouping increase’.(Hamilton, 2010). Consequently, average output per group member follows an inverted‐U shape curve, as drawn in many studies (Angulo et al., 2018; Clark & Mangel, 1986; Clutton‐Brock, 2016; Courchamp et al., 1999), with different names for the increasing returns: ‘inverse density effects’, ‘component Allee effects’ or ‘group augmentation effects’.

Another central idea, perhaps original, is that average cost per birth for a reproductive female declines as her number of births increases. Reproduction requires initial investments in reproductive organs, finding a mate, acquiring and preparing a breeding site and so on. As her number of births increases these initial costs are spread over more births, reducing their average cost. Additionally, her costs of brood care, defence and (for mammals) lactation increase less than in proportion to her number of births. And because even one birth may strongly diminish her ability to forage, this indirect cost per birth also declines as births increase. This reproductive female may reduce costs per birth still further by specialising her body for reproduction as with social insect or naked mole rat queens. Because of declining average costs per birth to a female, a group can achieve more births from its resources (e.g. food) by restricting reproduction to a single highly fertile female supported by others.

How could natural selection lead most females in a group to sacrifice their own direct fitness benefits while they support reproduction by another? Limiting reproduction in this way greatly increases the genetic relatedness of group members, which, for example, is high for naked mole rats (Faulkes 2021). Gains from cooperation in both production and reproduction, together with high relatedness, can provide indirect fitness benefits for non‐reproductives that facilitate evolution of these arrangements.

A large literature on reproductive skew focuses on the interests and behaviour of individual females: how does a dominant female get away with limiting the fertility of others? Why do not the subordinate females leave the group? Our approach is different. We suggest that reproductive skew can raise total reproduction in the group, enhancing group fitness. In standard reproductive skew notation (Nonacs & Hager, 2011), we suggest that k = k(p) (where k is total reproduction by the group and p is the subordinate's share of group reproduction). That is, we suggest that greater skew (smaller p) can increase total group reproduction (k).

We proceed in two steps, developing single‐sex models that ignore males. Analysis is based on constrained maximisation of fitness as is common in life history theory (Charlesworth, 1994). First, a simple numerical model illustrates how equilibrium group size is determined for a social species experiencing increasing then diminishing returns to numbers in production and scale returns in reproduction. It then illustrates how a population composed of such groups reaches an equilibrium size of group, number of groups, number of reproductives per group and density in an environmental area. Second, we develop a more abstract general model of selective forces driving the evolution of such a social species, now allowing for two kinds of reproductive limitation—lifetime and stage. We simulate a lineage's evolutionary trajectory from a solitary to a social species.

## COOPERATION IN PRODUCTION

Cooperation and group living can raise a species' ability to produce fitness‐enhancing outputs. Increasing returns could reflect division of labour, with castes (workers/soldiers) or without (sentinel duty/foraging), tasks requiring more members (carnivores hunting larger game (Smith et al., 2017) or Japanese honeybees ‘cooking’ a giant Asian hornet (Ugajin et al., 2012)), or information sharing. Larger group size also incurs costs requiring a larger territory with more costly defence, higher travel costs for foraging, greater difficulty recognising group members, more intra‐group competition, more complex coordination, contagion and wider sharing of gains. As group size grows, diminishing returns eventually replace increasing returns. The marginal return is the change in output when one member is added to the group. When the marginal return rises there are increasing returns; when it falls there are diminishing returns. Maximum average output per member occurs when the average rate equals the marginal rate.

## COOPERATION IN REPRODUCTION

In many social species—mammals (Smith et al., 2017), birds (Cockburn et al., 2017), insects (Hölldobler & Wilson, 2009), fish (Taborsky & Wong, 2017), shrimp (Hultgren et al., 2017) and other taxa—reproduction in a group may be restricted to a single female or subset of females. Fertility restriction in a group can take two basic forms: lifetime restriction or restriction to a stage of adult life. Lifetime restriction often involves a single reproductive female (e.g. many eusocial insects (Hölldobler & Wilson, 2009), naked mole rats (Smith et al., 2017), snapping shrimp (Hultgren et al., 2017), social thrips (Abbot & Chapman, 2017) or social fish (Taborsky & Wong, 2017)). Evolution is facilitated by strong intra‐group relatedness, achieved by having a single reproducer. Alternatively, every female reproduces during an adult stage, outside of which she is non‐reproductive. Each period some fraction of a group's females is at the reproductive stage while those outside that stage support their efforts. In some species, *younger* adults reproduce with help from older adults, as in humans and four species of toothed whales (Ellis et al., 2018). In other species, *older* females reproduce while younger females assist, as with African wild dogs (Creel & Creel, 2002). With stage restriction, all females do incur initial reproductive fixed costs (as described earlier) but can avoid maintenance costs and can reduce other costs by concentrating reproduction in one stage of adult life. With stage‐restriction, all females reproduce and potentially achieve direct fitness gains so indirect benefits and group relatedness are less necessary (Clutton‐Brock, 2002). Furthermore, support by non‐reproductives is often directed to kin, as with postreproductive human grandparents assisting their adult children and grandchildren (Hawkes et al., 1998; Hooper et al., 2015; Kaplan et al., 2009).

## POPULATION DYNAMICS AND EQUILIBRIUM

We will consider both individual and group reproduction, as well as the equilibrium number of groups and population density in an environment. Individual reproduction requires surviving births. Here, group level reproduction takes place through fissioning when a certain group size is reached, although group reproduction takes different forms in different species—for example, individual females may leave alone to establish new colonies. However, the basic forces in this specific illustrative model are more generally relevant. We here address what Clutton‐Brock (2016:83) describes as the ‘largely unexplored’ topic of how female sociality can affect ‘population demography and the regulation of population density’.

**FIGURE 1:**
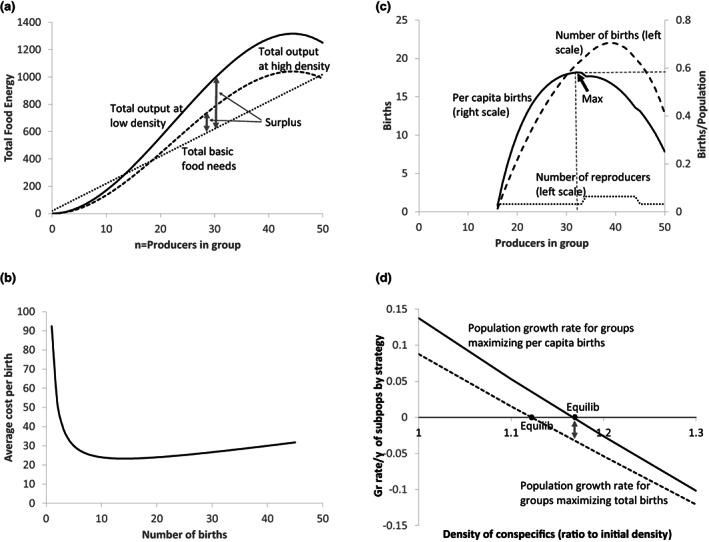
A social group produces surplus (a) that is converted into births by one or more reproductives (b) and (c). The conspecific population of members of these groups grows, raising density and reducing surplus, until a population equilibrium is reached where surviving births equal deaths in a representative group (d). Grey arrow indicates that the group maximising total births (dashed line) would decline at 0.03/y and go extinct when the group maximising per capita births (solid line) reaches its equilibrium density. (a) Output by density, consumption and surplus; (b) For a reproductive, cost per birth declines; (c) Total and per capita births and reproducers; (d) Pops grow until birth rate = death rate

Figure 1 graphs the functions in this illustrative model which incorporate key relationships and constraints faced by a hypothetical social species. The functions could in principle be estimated using field data of the sort gathered for African wild dogs (Creel & Creel, 2002), honey bees (Seeley, 2019) or social spiders (Yip et al., 2008). Empirical estimates for food production (usually capture of prey) are available for some species but are complicated by the role of season, foraging risks, energy expenditure, protection of kill and defence of territory.

The equations represented by the graphs are given in Box 1. Panels A‐C refer to a single representative group in the local conspecific population of which it is assumed to be a small enough fraction that we can ignore the effects of its changing numbers on population size and density. Panel D describes the dynamics of the whole local population assuming each constituent group is like this one except for reproductive goal.

BOX 1 **Numerical simulations for population dynamics and equilibrium, Figure 1a–d**

This box provides details about the calculations underlying Figure 1, panels a–d.

Figure 1a

For a given population density D, total output Y is a cubic function of number of producers, n, in a single representative group. At higher densities, output for each number of producers is reduced. The density effect of *D* ^−*0.7*^ assumes that output is a function of land available to the group with an exponent of 0.3. This specification is somewhat arbitrary but changing the density effect would not affect the qualitative results. Equation B1.1 gives the function used in Figure 1a,b.

(B1.1) $$Y=D^{-0.7}\left({\text{2n}}^{2}-.03n^{3}\right)$$

Per capita subsistence food need is 20, so total surplus is given by (B1.2). Upper case N is total group size including reproductives, so N ≥ n + 1. The initial density is 1. To illustrate the effect of density, output is also plotted for *D = 1.9*.

(B1.2) S=Y−20N

Figure 1b

A quadratic total cost function for births for a single reproductive female:

(B1.3) $$C=20+\text{3B}+.{\text{1B}}^{2}$$

Average cost is (B1.3) divided by B:

(B1.4) C/B=20/B+3+.1B

Figure 1c

For given group size and density *D = 1* surplus available for reproduction is given by (B1.2) and plotted in Figure 1a. Births generated from a given surplus are given implicitly by (B1.3) for a single reproductive female. To find births explicitly we solve the quadratic cost equation (B1.3) with different numbers of reproductives, 1, 2, 3 or more if necessary to find the number yielding maximum surviving births. For example, one reproductive requires 20 units of food for subsistence leaving *S‐20* for additional costs of reproduction. We seek the number of surviving births *B* that can be generated by a single reproductive female for given *S* and the birth cost function (B1.3):

(B1.5) $$S-20=.{\text{1B}}^{2}+\text{3B}+20\;0=.{\text{1B}}^{2}+\text{3B}+40-S$$

The quadratic equation gives:

(B1.6) $$B\left(S\right)=\left(-3\pm \sqrt{9-.4\left(40-S\right)}\right)/.2$$

Similar calculations for the case of two or three reproductive females are straightforward. With three reproductives available food per reproductive is *(S‐60)/3* and total births are three times the births per reproductive. Searching over number of producers and number of reproductives at each density reveals the maximum possible number of births at that density or alternatively the maximum birth rate (per capita births). The per capita birth line is found by dividing the number of births by the number of group members, which is number of producers on the horizontal axis plus the number of reproductive females (sometimes one, sometimes two).

Figure 1d

Assume the adult death rate is 0.4. For given density D we find how many producers and reproducers yield maximum per capita births using calculations like those for Figure 1c. This associates a maximum birth rate (per capita births) with each density D. Subtracting the death rate (0.4) gives the population growth rate associated with each density. This relationship is plotted as the solid line. When it is above zero, population grows and density D increases until it reaches equilibrium at 0 with density D = 1.66, marked by a dot. At lower densities the population grows; at higher densities it declines. The lower line (dashed) is similar but is based on maximising total surviving births rather than per capita. Figure 1c shows a larger group size will be chosen in this case with lower per capita births. Thus, this dashed line is lower in Figure 1d, and equilibrium is reached at a lower density of 1.12 marked by a dot. If both kinds of groups are present in the environment the ones maximising per capita births will prevail. At the higher equilibrium density of the per capita birth maximisers, the groups maximising total births will be unable to sustain themselves and will decline at a rate of 0.03 per year (shown by grey arrow), eventually going extinct.

Total output in Panel A initially displays increasing returns followed by diminishing returns as discussed earlier. Basic adult food needs (for survival) are proportional to number of producers plus reproducers as shown by the straight line. Total output minus total basic food needs is the surplus available to support the additional costs of reproduction. Groups with less than 16 producers or more than 53 would not be sustainable because food needs would exceed output. The number of producers that maximises total surplus occurs where the derivative of the output function equals per capita food needs. Beyond that size, each additional member raises output less than she raises total food needs. A number below this maximises per capita surplus where the derivative equals average output per producer.

The total output curve reflects the quality of the environment, which we assume is fixed, and depends on the density of conspecifics in this environment. The top output curve shown here is for the ‘initial’ density, call it D = 1. The lower output curve reflects a higher density with reduced surplus such that the same effort by each number of group members would yield less output.

Panel B shows how the average cost of a surviving birth for a single female initially declines as her fixed costs of being reproductive are spread over a larger number of births. We assume that eventually, the average cost per offspring begins to rise (Box 1 for details). Using total surplus generated by the group, we calculate the number of surviving offspring that could be generated by a single reproductive or by other numbers of reproductives and then select the number that would yield the most surviving offspring for the group.

Panel C shows the result of this calculation for initial density (D = 1) based on surplus generated by each number of producers from Panel A which is then combined with Panel B to find the corresponding number of surviving offspring for each number of producers. The number of reproductives generating the most surviving births is shown by the line at the bottom: a single reproducer for producers numbering 16–33, then 2 from 34 through 44 where total surplus is high (although per capita surplus is falling), reverting to 1 as total surplus falls. The dashed line gives the total number of surviving births, and the solid line gives the per capita number of surviving births (the average surviving birth rate). Maximum total offspring are achieved with 39 producers and two reproductives. However, offspring per female (the surviving birth rate) is maximised at 0.58/y with 32 producers and a single reproductive. So for density D = 1 the maximum attainable birth rate is 0.58. The inverted U‐shaped relation of total births to group size shown here has been found empirically for social spiders (Yip et al., 2008) and African wild dogs (Creel & Creel, 2002). The rising part of the surviving birth rate curve exhibits a so‐called demographic Allee effect in which individual fitness increases with group size or density. The rising output per member at low densities implicit in Panel A is a component Allee effect (Angulo et al., 2018).

Now consider adult mortality which we assume is a death rate of 0.40/y. The initial surviving birth rate of 0.58/y exceeds the death rate by 0.18/y so the population will grow rapidly and eventually fission, and other similar groups will also grow and fission at this density. Consequently, the overall population density will increase. As density rises the total surplus and birth rate in each group will decline and population growth will slow. Eventually, this process of growth, fissioning and declining surplus slows and stops as the birth rate approaches the death rate. This is the equilibrium point for total population size, density, number of groups and surviving birth rate. Panel D shows the maximum surviving birth rate at different population densities from D = 1 to 3, assuming the output curve (Panel A) shifts with density by a factor of D^−0.7^ (other specifications would yield similar results).

Group selection arises through differences in the relative fitness of social groups. But what is the appropriate measure of fitness and how does it depend on group structure and modes of reproduction (Charlesworth, 1994)? Is it fitness of the foundress, or frequency with which new colonies are formed (through fissioning, swarming or budding), or the growth rate of the population? In the context of our model, the three fitness measures appear to be equivalent, and detailed formal analyses of honey bees have found them to be so: ‘Finally, r is not only the growth rate of the number of queens (and colonies), but is also the growth rate of the total population’. (Al‐Khafaji et al., 2009) p.561.

In this setting, Panel D shows that natural selection will favour groups that maximise their surviving birth rate rather than their total number of surviving births. The ‘surviving births’ groups equilibrate at density D = 1.12 where the birth rate line crosses the death rate line. But at this density, the ‘surviving birth rate’ groups are still growing at 0.036/y. They continue to grow until reaching density D = 1.7. At this density, the population of the ‘surviving births’ groups would decline at 0.03/y and go extinct.

The optimal group size (maximising the growth rate of the group and hence the rate of fissioning) does not necessarily depend on the conspecific population density or may vary with it only modestly.

Through chance some group might find itself at an unsustainably low size. It might then grow by accepting new members from outside or by kidnapping young from neighbouring groups as with choughs (Heinsohn, 1991) and naked mole rats (Braude & Hess, 2021).

## A CASE STUDY: *LYCAON PICTUS* (AFRICAN WILD DOGS)

African wild dogs (AWD) have been closely studied; here we draw chiefly from Creel and Creel (2002) henceforth CC who studied packs in Selous Reserve. AWD live in packs of 2–27 adults and yearlings (CC:4) that are typically formed by a set of closely related females and closely related males (often litter mates, with males unrelated to the females) (CC:197). They hunt cooperatively and are obligate cooperative breeders with a single breeding female who hormonally suppresses the other females. Males and non‐breeder females hunt and regurgitate food for the alpha and pups and share in guarding the pups. The coefficient of relatedness among packmates averaged 0.25–0.35 (CC:6).

**FIGURE 2:**
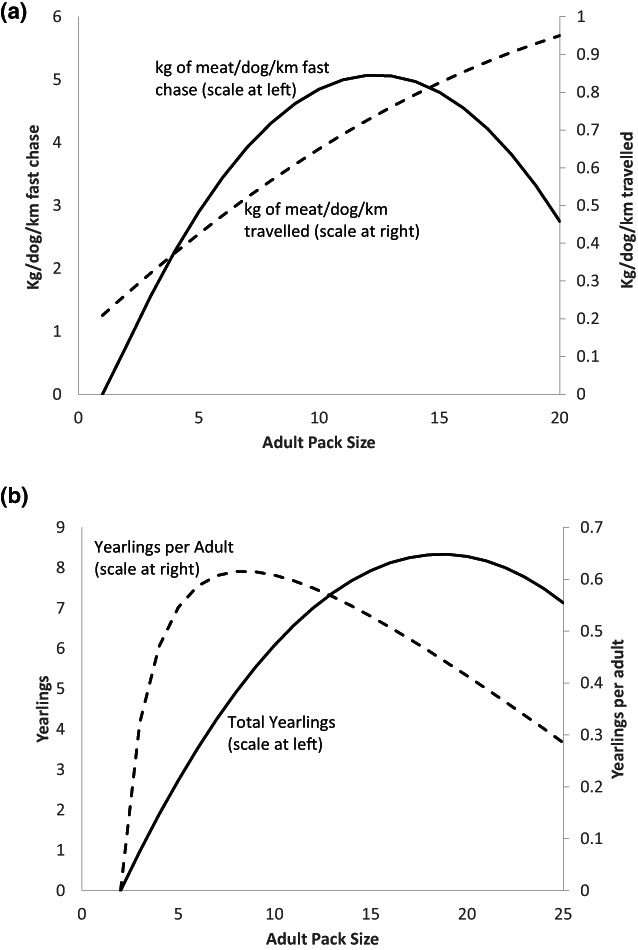
Increasing and diminishing returns to pack size in production and reproduction for African wild dogs (Creel & Creel, 2002). (a) Two measures of cost‐adjusted hunting success by adult pack size: kg of meat per dog per km travelled and per km of fast chase. (b) Source: Both curves are plotted from fitted quadratic equation reported by Creel & Creel, 2002:88–89. Selous data. 11 pack years of observation, 404 kills, 905 hunts. (c) Total annual yearlings and yearlings per adult, by adult pack size (data from Selous, Kruger and Serengeti). (d) Source: Total yearlings plotted from fitted quadratic equation reported by Creel & Creel, 2002:172. Yearlings per adult calculated by authors from same fitted quadratic and adult pack size. Data from 57 pack years of observation.

Food acquisition by hunting is initially subject to increasing returns to adult pack size (CC:89–90) as shown in Figure 2. One measure, kg of meat/dog/km travel, rises throughout the observed pack size range up to 20 without exhibiting diminishing returns. A different measure, kg of meat/dog/km fast chase, shows strong increasing returns peaking at 12 dogs followed by diminishing returns. CC:95 suggest that no single ecological variable ‘is likely to explain variation in fitness across pack sizes’. The impractical but conceptually correct approach would be to ‘identify all the variables likely to affect fitness, quantify each as a function of groups size, convert them to a common currency (or establish weighting factors for the impact of each variable on fitness)….’(CC:95) Such variables for AWD include increasing likelihood of dispersal as the breeding que lengthens; defence of a kill; defence of territory; predation avoidance particularly for offspring; degree of relatedness in pack (CC:96).

The breeding alpha female produces one litter per year after 70 days gestation (CC:159,167) followed by 2–3 months in a den. In the Creels’ study no pack smaller than 5 was able to raise pups successfully. The number of yearlings (surviving births) rises strongly with the size of the adult pack peaking at size 16 to 19, and then declines (Figure 2b).

Our interpretation is that the larger pack size provides more meat for the alpha female and her pups, enabling her to have higher fertility, and the larger pack size also helps her protect the pups thereby raising their survival to become yearlings (although survivorship is not in our model). Our analysis indicates that it is yearlings per adult (the surviving birth rate of the pack) rather than the total number of yearlings that is most relevant for fitness of the group and its members. When we calculated this birth rate we found it peaked at size 8–9 adults (Figure 2b). CC:294–5 simulated the population growth rate incorporating relations to pack size of hunting, reproduction and survival, finding that the growth rate peaked at pack size of nine adults, consistent with the peak birth rate per adult dog in Figure 2b and with the actual average pack size of 8.9 adults in Selous (CC:60). This is consistent with our earlier analysis (Figure 1c). C&C:294 say ‘It is not surprising that a decrease in pack size would reduce the population's growth rate, but *a priori* it is less obvious that an increase in pack size would have the same effect’, but that is what our analysis predicts (Figure 1c).

Because all the AWD females are physiologically equipped for breeding, the suppressed status of non‐breeders does not save fixed costs of growing reproductive organs. However, a pregnant or lactating dog is ineffective at hunting (CC:72). If every female were to breed, then the hunting effectiveness of all females would be reduced and the food acquired for any given pack size would likewise by reduced. We suggest that group fitness and average individual fitness of group members are both enhanced by limiting reproduction to a single female so that the others can hunt. This is in line with the ‘declining average costs of fertility’ theory advanced in the main text—the breeding female spreads the costs of her foregone hunting over all her pups, an average of 7.9 in Selous (CC:159) and up to 21 (CC:167). With all reproduction concentrated in the single alpha female rather than each female having a small number of pups it is possible for all pack members and pups to be better fed, or for the dogs to expend less time and energy hunting, or for the alpha to have an additional pup or two, raising the fitness of the group and its individual members.

In (Creel & Creel, 2002) and (Woodroffe, 2011) average pack size remains stable while number of packs may grow, consistent with the optimal pack size approach.

## EVOLUTIONARY OPTIMUM AND TRAJECTORY

The preceding discussion presupposed the existence of a social species. Now, we consider how and why forces of natural selection could lead a lineage to evolve from an originally solitary species towards a social one with cooperative breeding. First, some notation and preliminaries.

### The model

A group has *N* coresiding females each of whom has one unit of time over her lifetime. Of *N* coresiding females a proportion *p* are reproducers (who may also produce part‐time) and *1‐p* are non‐reproductive producers. For an individual, becoming able to reproduce requires a fixed investment of time *t*, *0 ≤ t ≤ 1* (since an individual's lifetime = 1, *t* is the proportion of her lifetime), to grow and maintain reproductive organs, find and select a mate and breeding site, etc. Individuals who have made this investment are called ‘reproducers’ or ‘reproductives’. To reproduce, a reproductive must additionally allocate variable time *x*, *0 ≤ x ≤ 1‐t*, (again a proportion) for pregnancy, lactation, offspring care, guarding, maternal recovery, etc. This leaves reproductives with time for production *1‐t‐x*, 0 *≤ 1‐t‐x ≤ 1‐t*.

The total number of equivalent full‐time producers is *n* where

(1) $$N=\left(1-p\right)N+\mathrm{pN}\left(1-t-x\right)$$

The total output Y generated by the group is a function *g(n)* of this number or

(2) $$Y=g\left[N\left(1-\mathrm{pt}-\mathrm{px}\right)\right]$$

(As with the earlier model plotted in Figure 1a, *g* depends on the conspecific density and the quality of environment, but here we take these as given.)

Each group member requires basic energy *s* to survive and function. Each reproductive female devotes additional energy *m* to reproduction. The energy budget constraint for the group equates total energy output to total energy use:

(3) $$g\left(N-\mathrm{Npt}-\mathrm{Npx}\right)=\mathrm{Ns}+\mathrm{Npm}$$

The number of surviving births per reproducer is *b*,

(4) $$b=f\left(m,x\right)$$

*f(m,x)* is assumed to be homogeneous of degree 1, meaning that if time and energy inputs increase proportionately (e.g. double), then surviving births increase in the same proportion. Here, food produced by others contributes to *m*, while the time of others cannot be substituted for maternal time *x*—a simplifying assumption which is wrong for some social species but which could readily be altered. The total surviving births in the group is *pNf(m,x)*. As discussed in the previous section, natural selection will favour groups with the highest average rate of surviving births per group member, π, and also favour individual members of these groups relative to members of others, conditional on the degree of relatedness within the group and size of direct and indirect fitness gains

(5) $$\pi =\mathrm{pf}\left(m,x\right).$$

As noted earlier, this fitness criterion gives the same results as the rate of increase of groups or the rate of increase of the foundress, at least in cases we have examined.

After some substitution and simplification including solving out the variable *m*, the optimisation problem becomes

$\max_{N,p,x}\;\pi \equiv \mathrm{pf}\left(\frac{g\left(N-\mathrm{Npt}-\mathrm{Npx}\right)-\mathrm{Ns}}{\mathrm{Np}},x\right)$ subject to the bounds:

(6) N≥1,1/N≤p≤1,0<x≤1−t.

If the optimum for a variable occurs on a boundary it is a ‘corner solution’. An optimum occurring within a variable's boundaries is an ‘interior solution’. For an interior solution, the derivative of π with respect to the variable must be zero.

Analysis of the derivatives yields important results:

(7) $$\frac{\partial \pi }{\partial n}=\frac{f_{m}}{n^{2}}\left[ng^{\prime }\left(n\right)-g\left(n\right)\right]\;\mathrm{so}\;\frac{\partial \pi }{\partial n}=0\Rightarrow g^{\prime }\left(n\right)=\frac{g\left(n\right)}{n}$$

This is the same condition given in the preceding section: per capita output is maximised when the average rate *g(n)/n* equals the marginal rate *g՛(n)*. The optimal *n* is always an interior solution since there is no upper bound on *N > n* although eventually production experiences diminishing returns such that a solution to (7) always exists.

After calculating the fitness derivatives for *x* and *p* we find the following:

(8) $$\frac{\partial \pi }{\partial x}=0\Rightarrow \frac{\partial \pi }{\partial p}<0;\;\frac{\partial \pi }{\partial p}=0\Rightarrow \frac{\partial \pi }{\partial x}>0$$

Equation (8) says there cannot be an interior optimum for both *x* and *p* at the same time. If an optimum has *x* at an interior value with derivative 0, then the corresponding *p* must be at its lower boundary of *1/N* (a single reproductive) with a negative derivative. Because it is at its lower boundary, a lower value of *p* cannot evolve although doing so would raise fitness. This corresponds to the case of a single female reproductive who divides her time optimally between reproduction and production. Examples are African wild dogs (Creel & Creel, 2002), naked mole rats (Smith et al., 2017), social insects (Hölldobler & Wilson, 2009), social thrips and aphids (Abbot & Chapman, 2017), social fish (Taborsky & Wong, 2017), social birds (Cockburn et al., 2017).

Alternatively, if an optimum has *p* at an interior value where its fitness derivative is 0, then *x* must be at its upper boundary *1‐t* with a positive derivative, indicating that a higher value of *x* cannot evolve although doing so would raise fitness were it possible. This corresponds to the case where every adult female is reproductive for a stage of her life (conditional on surviving to a sufficient age), but during that stage, she is fully occupied with reproduction and is only productive outside of that stage. This would be an extreme case, but examples tending in this direction are humans (Hooper et al., 2015), some toothed whales (Ellis et al., 2018), African wild dogs (Creel & Creel, 2002), meerkats (Smith et al., 2017) and birds with helpers (Cockburn et al., 2017). Further details on these results are given in Box 2.

BOX 2 **Equations for the optimum and evolutionary trajectories f or a social lineage**

At an interior optimum, the partial derivatives of π with respect to *n, p* and x must each be 0. The first of these, ∂π/∂n, was given earlier. The other two are as follows:

(B2.1) $$\frac{\partial \pi }{\partial p}=f-f_{m}\left[\left(x+t\right)g’+m\right]$$

An increased proportion of reproducers tends directly to raise surviving births but indirectly to reduce them by reducing total group time for production and requiring additional total energy for reproduction. If *t = 0* with no fixed cost of reproduction, then any value of *p* would be optimal including *p = 1*.

(B2.2) $$\frac{\partial \pi }{\partial x}=p\left(f_{x}-f_{m}g^{\prime }\right)$$

Greater *x* directly raises surviving births but indirectly reduces them by reducing foraging time and energy for reproduction.

Analysing these derivatives yields the important result given in (8).

Turning to the simulated dynamic trajectories and isofitness plots, the output function is specified as:

(B2.3) $$g\left(n\right)=A\left(\frac{1}{1+q^{-n+n_{0}}}-\frac{1}{1+q^{n_{0}}}\right)$$

A shifts the curve up or down. *n*_*0*_ shifts the curve left or right. *q* makes the curve steeper.

Surviving births per capita, π, are given by the following equation:

$\pi =\mathrm{pf}\left(m,x\right)$; $f\left(m,x\right)={\mathrm{Bx}}^{\beta }m^{1-\beta }$; $\pi =\mathrm{pB}{\left(\frac{g\left(n\right)-\mathrm{Ns}}{\mathrm{Np}}\right)}^{1-\beta }x^{\beta }$.

(B2.4) $$\pi =\mathrm{pB}{\left(\frac{g\left(N-\mathrm{Npt}-\mathrm{Npx}\right)-\mathrm{Ns}}{\mathrm{Np}}\right)}^{1-\beta }x^{\beta }.$$

### Why lifetime vs stage‐based fertility limitation?

Whether a lineage's optimum has interior *x* or *p* depends on its specific functions *f* and *g* and values of *s* and *t*. Here we focus on the reproduction function (Binford, 2001), *b = f(m,x)*, and consider a specific functional form: *b = αm* ^β^ *x* ^*1*−β^. A larger β indicates that food *m* is relatively more important than maternal time *x* in reproduction, so that reproduction is ‘food intensive’ while smaller β indicates it is more ‘time intensive’. For example, when reproduction involves a large litter size (most birds, fish and insects) it is bound to be more food intensive since scale economies in per capita care time do not reduce the need for food per capita. When reproduction involves a singleton birth (humans, great apes, whales, King penguins) it will be more time intensive. In the food‐intensive case, the productive females (subordinates or workers) can supply food to a single reproductive female making it possible for her to have many surviving births, so the outcome with a single reproductive is more likely. In this case, she would then optimally allocate her time between reproduction and production. She might reproduce full time (honeybees) or only part time (African wild dogs). In the time‐intensive case, available maternal time (*1‐t*) puts an upper limit on the number of surviving births for a single reproductive female, so the stage‐limited outcome with many reproductive females is more likely. For some level of food or time intensity, an optimal outcome at the intersection of the *x* and *p* boundaries is also possible.

These points depend on the earlier assumption that supporters can supply food to the reproductive female but cannot substitute for her time. However, even if supporters can substitute for some of her time, the outcome could be the same if there were some component of the reproductive's time that could not be substituted, such as the time required by a honey bee queen to generate and lay her multitude of eggs, or the time pregnant of a female mammal.

## SIMULATED EVOLUTIONARY TRAJECTORIES TO A SOCIAL OPTIMUM

A simulation based on specific functional forms and parameter values will help clarify the meaning of these abstract results. We use different values of the β parameter in the reproduction function just discussed, together with other functions and parameter values (see Box 2).

### Evolutionary trajectories

**FIGURE 3:**
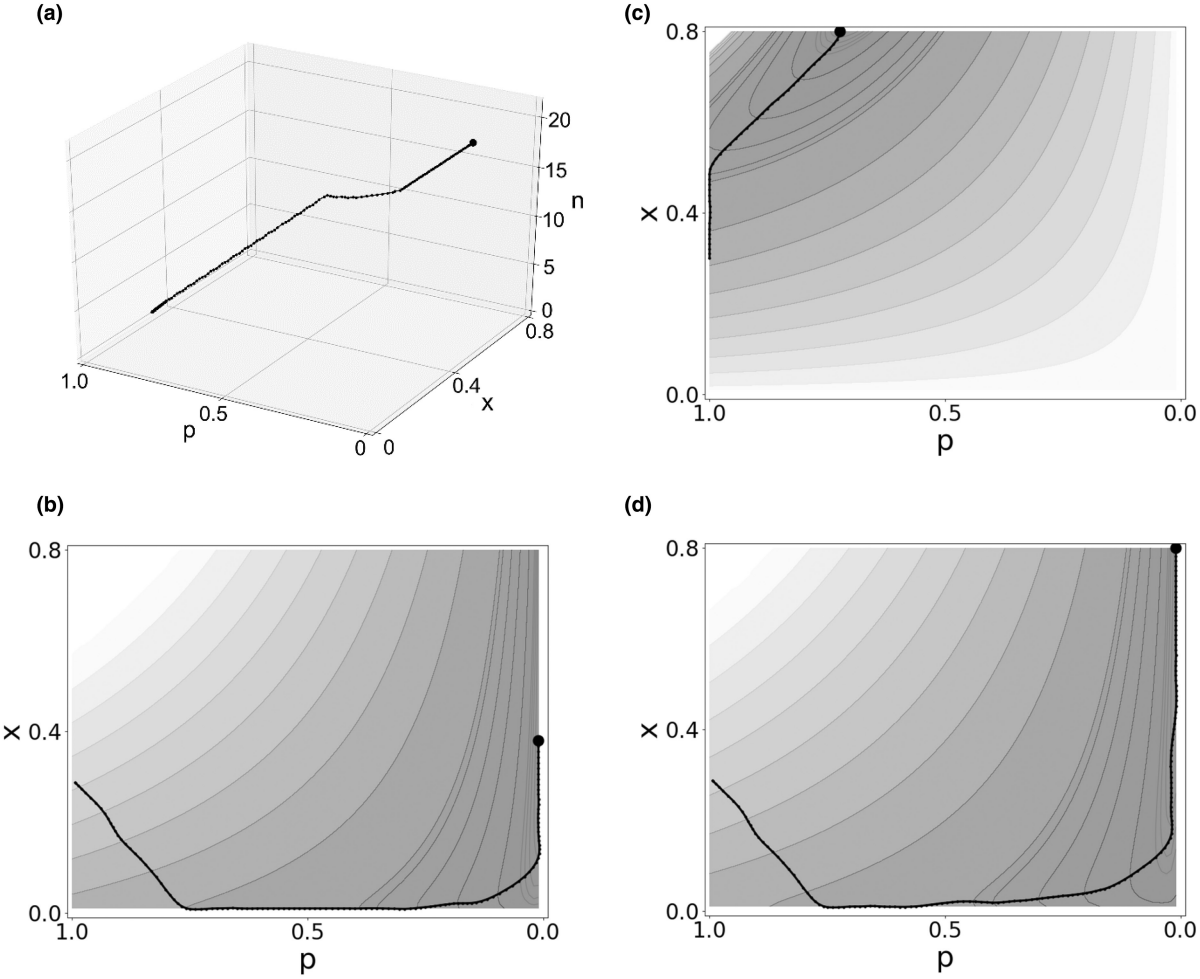
Fitness isoquants, optimal social arrangement and evolutionary trajectories for lineages with differing time intensity of reproduction but otherwise same parameters. (In all panels t=.2,s=100). Optimum is indicated by black dot on all panels. Panel (a) plots three‐dimensional (3D) evolutionary trajectory through box for food‐intensive case. Panels b, c and d plot 2D versions of trajectories projected to bottom of box, not showing n. b, c and d also plot 2D fitness isoquants for *p* and *x*, which pass through all points in the plane that have the same level of fitness. Areas with darker shading indicate higher fitness. Evolutionary trajectories move in the direction of the greatest increase in fitness, crossing each isofitness contour at a right angle. b plots the same food‐intensive case as A, converging to corner solution with single reproductive (*p = 1/N*). c plots time‐intensive case converging to corner solution with many reproductives each reproducing full time (*x = 1‐t*). d plots the intermediate case converging to intersection of boundaries of *x* and *p*, with *x = 1‐t, p = 1/N*. (a) 3D Food intensive (*β* = 0.995), Optimum: n* = 21.47, *x* = 0.48, *p* = 1/(n* + 1) = 0.045. (b) Food intensive (*β* = 0.995), 2D version of A with same optimum. (c) Time intensive (*β* = 0.25), Optimum: n* = 21.47, *x* = 1‐*t* = 0.8, *p* = 0.73. (d) Intermediate intensity (*β* = 0.99), Optimum: n* = 21.47, *x* = 1‐*t* = 0.8, *p* = 1/*N* = 0.045

For convenience, we assume that the functions governing production *g* and reproduction *f* remain stable as the lineage evolves through a sequence of species as it converges to its optimal social arrangement. Imagine a three‐dimensional box with bounded values of N = n/(1‐pt‐px), *p* and *x* on the axes (n is bounded only below). Each point on or inside the box corresponds to some level of fitness π given by (6). Figure 3a plots an evolutionary trajectory for a lineage beginning with a solitary species (*N = 1, n = 0.3, p = 1, x = 0.3*) at the lower left end of the trajectory. This species evolves to live in larger groups (*N > 1*) with reproductive limitation (*p < 1*), eventually arriving at a social species with a single reproductive female who spends about half her time producing, at the upper right end of the line. The initial solitary species is not optimal, but is constrained to suboptimal values of N, p and x by initial traits such as mating behaviour, degree of relatedness with nearby others, inability to cooperate, inability to tolerate others, non‐progressive feeding and so on. Natural selection will tend to modify these traits to raise fitness by taking better advantage of the cooperative possibilities implicit in f and g, leading to the evolution of new species with traits more favourable to social life. Although the trajectory is plotted as a smooth line in 3a, we imagine that it passes through a series of discrete species forming steppingstones from the ancestral solitary species towards the species with optimal social organisation and greatest fitness. Steps would feature preadaptations such as monogamy, extended offspring care, non‐dispersal, allocare, group member recognition, cooperation in production and hormonal suppression of other females' reproduction. Phylogenetic trajectories of this general sort have been diagrammed (Hunt & Toth, 2017; Smith et al., 2017). Simulations show convergent evolution in the sense that if parameter settings are the same, then lineages starting from different initial points converge to the same optimal *n*, p and x. However, if the initial starting points have different parameter values, then they progress to different evolutionary optima.

### Fitness isoquants and optima

Although *n, N, p* and x evolve jointly in three dimensions as shown in Figure 3a, visualisation and interpretation of the trajectory are difficult. Figure 3b–d simplify the graph by ignoring *n* to focus on trajectories for *p* and *x* alone, projected onto the two‐dimensional plane on the bottom of the box in Figure 3a. Each panel shows fitness isoquants as thin curved black lines and shading, with darker areas indicating higher fitness. Panels also show evolutionary trajectories as heavy black lines, always moving in the direction of the greatest increase in fitness, crossing each isofitness contour at a right angle. Each trajectory ends in an optimum indicated by a solid black circle. Panels b, c and d represent three different cases generated by varying just one aspect of the model: the food vs time intensity of reproduction, indicated by β in the birth function. Each panel shows trajectories from the same starting point at which a solitary female (*p = 1*) devotes 30% of her time to reproduction and 70% to production (*x = 0.3*), but the lineages converge to different optima, each with n* = 21.7. By (8) the optimum must be on an edge (boundary) of the box.

- For a high value of *food* intensity (3A and 3B), the optimum occurs at the right boundary for *p = 1/N* close to 0 with a single reproductive female who produces part time (*x = 0.48*) but is mostly provisioned by others (e.g. African wild dog).
- For a high value of *time* intensity (3C), the optimum occurs with stage‐based reproductive restriction at the top boundary with reproductive time *x* at its maximum (*x =* .8=1−t) for those females currently in reproductive stage who make up *p = 73%* of total females (e.g. human).
- In between (but still with relatively high food intensity) is an optimum at the intersection of the *p* and *x* boundaries with a single fully specialised reproductive female (*p = 1/N, x = 1‐t*) (e.g. eusocial insects).

## DISCUSSION

### General

We have focused on the benefits of sociality, group living and cooperative breeding, whereas most of the theoretical literature has focused on how cooperative and altruistic behaviour could evolve even though they involve sacrifice of individual direct fitness. Restriction of *lifetime* reproduction to a single reproductive female brings a high degree of group relatedness and corresponding indirect fitness benefits, facilitating its evolution. Restriction of reproduction to a *stage* of adult lifetime for all females can increase the expected direct fitness of each female, again facilitating evolution. Direct benefits alone may in some cases be a sufficient basis for social behaviour (Clutton‐Brock, 2002). For example, of 213 species of cooperatively breeding birds, 30% nest in mixed kin/non‐kin groups and 15% nest primarily with non‐kin (Riehl, 2013).

We suggest that increasing returns in food production and reduced energetic costs of reproduction through reproductive skew are two fundamental drivers of the evolution of social organisation in many taxa, but the model and ideas apply more generally. Production of safety through sentinels and defence plausibly involves first increasing then diminishing returns to group size and similarly for thermal regulation, territory defence and other group benefits. Reproductive limitation plausibly reduces costs when a single female lactates, guards and cares for many offspring simultaneously. Empirical studies illustrate many of these points, yet field studies of increasing and diminishing returns remain rare for production and largely absent for reproduction. Nor has the time versus food intensity of reproduction been studied. These areas are ripe for research. Cross‐species analyses of social behaviour yield useful generalisations, but it can be difficult to see the bigger picture (Clutton‐Brock, 2002; Rubenstein & Abbot, 2017b).

Should both lifetime and stage‐restricted reproduction qualify for the term ‘eusocial’? Some say yes for the case of humans and other stage‐restrictors, noting that ‘**…**the distinct classes of sterile helper and reproductive that occur in women occur within, rather than among, individuals’ (Foster & Ratnieks, 2005). The idea of a more inclusive eusocial ‘continuum’ is appealing (Sherman et al., 1995), but implementation based on lifetime reproductive skew would rule out species with stage‐segmented restriction such as humans, since all females reproduce during that stage. However eusociality is defined, we hope that the distinction between lifetime and stage restriction and its suggested connection to litter size may be of some use in clarifying the issues.

### Does reproductive skew raise group fitness?

The literature on reproductive skew builds on a basic model for two females who would have some reproductive fitness outcome as solitary breeders and alternatively as cooperative breeders, where both outcomes reflect their relatedness and indirect benefits. The sum of their fertilities when they breed cooperatively is k times the sum of their individual fertilities, and a share *p* of this summed fertility accrues to female a and share *1‐p* to female b (*p* has different meaning in our model). With this basic setup, the range of possible outcomes is analysed and the stability of these in the face of dispersion is assessed. In our different framing *k* depends on *p* : *p* determines the fitness gains that arise from cooperative breeding. We are particularly interested in the case where *p = 0* and *1*‐*p = 1*, that is singular cooperative breeding. There are gains in this case for two reasons. First, with *p = 0* non‐reproductive females are freed from reproductive tasks which would limit their ability to forage and perform other tasks related to acquiring food, safety and defence, so the surplus available for group reproduction would be greater. Second, with *p = 0* non‐reproductive females do not need to invest resources in building and maintaining reproductive organs, costly mating or guarding and lactating a wastefully small number of offspring. The other female is then able to specialise fully in growing a highly specialised reproductive body as in the case of the honeybee queen or naked mole rat queen. For this reason, in cooperative breeding the outcomes will typically be a singular breeder, consistent with Lukas and Clutton‐Brock (Lukas & Clutton‐Brock, 2012): ‘In cooperative breeders, a single female monopolises reproduction and is responsible for over 90 per cent of breeding attempts (*n* = 26 species, median =100%, range 88–100%); species where reproduction is shared between plural breeding females were notably absent’.

If each female attempts to maximise her individual direct reproductive fitness then the 0–1 outcome would be unattainable (except perhaps through delayed dispersal in the face of its costs and risks) and the analysis in the reproductive skew literature would apply. However, if the females have acquired a mutation that allocates all reproduction to an alpha female based on a lottery at birth (honeybees) or on her age (African wild dogs), then their average reproductive fitness will be far higher due both to the fitness gains described above and due to the indirect fitness gains due to the higher relatedness resulting from their sisterhood.

### Does this theory apply to humans?

A single‐sex model for females must abstract from many important aspects of human sociality since for humans, males play an unusually important role. An analysis of data from 10 contemporary hunter‐gatherer societies (Kaplan et al., 2000) found that males produced 68% of all calories and 97% of surplus calories (i.e. net of producer's own consumption). However, if we average male and female production and consumption schedules at each age then the general pattern is in line with the theory. For example, Tsimane net nuclear family production (by parents and their children minus family consumption) remains substantially negative until the parents' mean age reaches 40, after which family net production is positive until parents reach their late 70 s or beyond, with surplus production transferred to younger group members (Hooper et al., 2015). We view our theory as consistent with and building on the theory of (Kaplan et al., 2009) on the evolution of human social organisation and also consistent with the Grandmother Hypothesis of (Hawkes et al., 1998) (in a single‐sex model these are similar), although these theories are far more detailed than ours, which is more formal, abstract and general. Our theory also places more emphasis on returns to group size in production and on reduced cost of reproduction by limitation to a subset of females.

Regarding increasing returns to number of producers in human hunter‐gatherer groups, (Binford, 2001) provides a meta‐analysis of ethnographic data on production in relation to group size. Key advantages of larger size arise from division of labour for childcare, hunting and gathering. Other things equal, key costs of larger size arise from the more frequent need to relocate camp to new foraging areas to avoid a need to travel over greater distances to forage. Binford does not estimate output in relation to group size, nor do we know any other study that does.

Regarding fitness gains from menopause and specialisation of reproductive and productive roles, postmenopausal women save energy relative to premenopausal. The menopausal state is 5–8% less energetically costly than the reproductive state because it avoids the costs of menstruation and of rebuilding the uterine lining every month (Hodson et al., 2014; Poehlman, 2002; Strassmann, 1996). In addition, incremental time needed for offspring care and lactation is far greater for the first offspring than for subsequent ones.

On the production side, reproductive age Tsimane females aged 20–45 averaged about 55% of the net production by older females (age 45–70) (Hooper et al., 2015). ‘…younger women are compromised in their food production by intensive childcare. Young babies are easiest to care for at a home base…. This trade‐off between food production and childcare may render the extra food provided by older women especially important’ (Gurven & Kaplan, 2006). Similarly, ‘older females may gain greater fitness by helping their adult daughters than by carrying additional, riskier pregnancies themselves’ (Hawkes et al., 1998).

### Longitudinal and cross‐sectional interpretations

Individuals live lives longitudinally but social interactions across ages occur cross‐sectionally each period. Reproductive fitness is a longitudinal individual trait but it is affected by social life. In the theory advanced here, becoming reproductive has a fixed lifetime cost t leading to lifetime specialisation as a reproductive or non‐reproductive. But in reproductive restriction by stage, every female may be a lifetime reproducer and therefore must incur the cost of becoming reproductive, although in cross‐section only a fraction *p* of females are in a reproductive stage at any given time. The mathematical theory did not involve age or generation, which would have greatly complicated it (Chu & Lee, 2013). Therefore, we can interpret it longitudinally in the lifetime case and cross‐sectionally in the stage‐limited case. In the lifetime case, *t* and *p* are lifetime variables, as described earlier. In stage‐limited reproduction, each female must bear initial costs of building organs. Now t can be interpreted as a cost of being in the reproductive state during the reproductive stage. The mathematical results can be interpreted in either way.

### Some testable hypotheses

1. In many cooperatively breeding groups, there are initially increasing and eventually diminishing returns to group size for an appropriate summary measure of weighted contributions to non‐reproductive aspects of group fitness such as food, safety, thermoregulation, range acquisition. Exceptions could arise if increasing returns or decreasing average costs in reproduction were sufficiently great in themselves (see points 2 and 3 below).
2. There are significant fixed costs for becoming reproductive, leading to diminishing average costs per birth as number of births to a given female increases.
3. There are diminishing incremental costs per birth as number of births per female increases due to economies of scale in key postbirth aspects of reproduction such as time costs of lactation for mammals or offspring care more generally.
4. The ratio of food inputs to time inputs per birth (or in total for lifetime or a breeding season) for a reproductive female is higher for cooperative breeders with a single reproductive female than for cooperative breeders in which each female reproduces in one life stage but is a non‐reproductive supporter in a different stage.

We believe that the central idea in this study, that reproductive limitation and concentration can lead to fitness gains through economies of scale in reproduction, leads to important theoretical insights and points to new topics for empirical research.

## AUTHOR CONTRIBUTIONS

Lee and Chu both contributed to modelling, analysis and drafting of this paper. Chu had primary responsibility for the sections on Evolutionary Optimum and on Simulated Evolutionary Trajectory. Lee had primary responsibility for all other sections.

### PEER REVIEW

The peer review history for this article is available at https://publons.com/publon/10.1111/ele.14157.

## Data Availability

No data are used in this theoretical paper.
